# TRAF6 Promotes Myogenic Differentiation via the TAK1/p38 Mitogen-Activated Protein Kinase and Akt Pathways

**DOI:** 10.1371/journal.pone.0034081

**Published:** 2012-04-04

**Authors:** Fang Xiao, Haixia Wang, Xinrong Fu, Yanfeng Li, Zhenguo Wu

**Affiliations:** Division of Life Science, State Key Laboratory of Molecular Neuroscience, The Hong Kong University of Science and Technology, Clearwater Bay, Kowloon, Hong Kong, China; University of Texas Health Science Center at Houston, United States of America

## Abstract

p38 mitogen-activated protein kinase (MAPK) is an essential kinase involved in myogenic differentiation. Although many substrates of p38 MAPK have been identified, little is known about its upstream activators during myogenic differentiation. TRAF6 is known to function in cytokine signaling during inflammatory responses. However, not much is known about its role in myogenic differentiation and muscle regeneration. We showed here that TRAF6 and its intrinsic ubiquitin E3 ligase activity are required for myogenic differentiation. In mouse myoblasts, knockdown of TRAF6 compromised the p38 MAPK and Akt pathways, while deliberate activation of either pathway rescued the differentiation defect caused by TRAF6 knockdown. TAK1 acted as a key signal transducer downstream of TRAF6 in myogenic differentiation. In vivo, knockdown of TRAF6 in mouse muscles compromised the injury-induced muscle regeneration without impairing macrophage infiltration and myoblast proliferation. Collectively, we demonstrated that TRAF6 promotes myogenic differentiation and muscle regeneration via the TAK1/p38 MAPK and Akt pathways.

## Introduction

Vertebrate skeletal muscle differentiation is regulated by multiple factors and intracellular signaling pathways [1]–[3]. The myogenic regulatory factors (MRFs) consisting of MyoD, Myf5, myogenin, and MRF4, and the myocyte enhancer factor 2s (MEF2s) consisting of MEF2A, MEF2B, MEF2C, and MEF2D, are two best-characterized families of transcription factors controlling the myogenic differentiation program [4]–[6]. In muscle cells, MRFs form heterodimers with gene E2A products (i.e., E12 or E47). Together, they specifically bind to a consensus DNA sequence called an E box [4]. MEF2 proteins form homo or heterodimers among themselves and the dimers bind to a consensus A/T-rich sequence called a MEF2 site [6], [7]. The activities of MRFs and MEF2s are in turn controlled by multiple intracellular signaling pathways that include the phosphatidylinositol 3-kinase (PI3K)/Akt- and the p38 mitogen-activated protein kinase (MAPK)-mediated pathways. The PI3K/Akt-mediated pathway is mainly activated by insulin-like growth factors (IGFs) and is known to transcriptionally regulate the expression of the *myogenin* gene [8]–[11]. Downstream of Akt, Foxo1a was implicated as a key transcription factor negatively involved in myogenic differentiation [12], [13]. In addition to the PI3K/Akt pathway, the p38 MAPK pathway is also known to promote myogenic differentiation via multiple mechanisms [14], [15]. p38 MAPK directly phosphorylates MEF2 and enhances its transcriptional activity [16]–[18]. p38 MAPK also phosphorylates E47, promotes its interaction with MyoD, and enhances the transcriptional activity of MyoD/E47 [19]. In addition, p38 MAPK could also phosphorylate BAF60 and facilitates the recruitment of the SWI/SNF complex to the promoters of muscle-specific genes [20]. Several isoforms of p38 MAPK, including p38α, p38β and p38γ, are known to participate in myogenic differentiation [21], [22].

Upstream of p38 MAPK, MKK3 and MKK6, two p38-specific MAPK kinases (MAP2Ks), are known to be involved in myogenic differentiation [17], [23]. Recently, TAK1, a MAP2K kinase (MAP3K), was also implicated in myogenic differentiation [24]. TAK1 plays a critical role in pro-inflammatory cytokine (e.g., interleukin-1β, tumor necrosis factor α) and toll-like receptor (TLR)-mediated signaling pathways [25]–[27]. In mammalian cells, TAK1 associates with TAB1/TAB2/TAB3 and is activated by TRAF6 in a Lys63-linked polyubiquitin chain-dependent manner [25], [26]. TRAF6 is a member of the TRAF family proteins that mainly function in IL-1R/TLR-mediated signaling pathways [26], [28]. In TRAF6-mediated signaling pathways, ubiquitination plays a very important role [25], [29]. Together with Ubc13 and Uev1A, TRAF6 catalyzes the synthesis of lysine-63 (K63)-linked polyubiquitin chain [29]. TAK1 is activated in a polyubiquitin and TRAF6-dependent manner [29]. Unexpectedly, unanchored K63-linked polyubiquitin chains were found to be sufficient in activating TAK1 in vitro [30]. In the TAK1/TAB complexes, TAB2 binds preferentially to K63-linked polyubiquitin chains, resulting in autophosphorylation of TAK1 at S187 and its subsequent activation [31]. Activated TAK1 phosphorylates and activates MKK3/MKK6, which in turn activate the p38 MAPK pathway. TAK1 also activates the NF-κB pathway by directly phosphorylating and activating IκB kinase (IKK) which in turn phosphorylates IκB and induces its ubiquitination and degradation by the proteosome-dependent degradation machinery [29]. Recently, TRAF6 was also found to catalyze direct Akt ubiquitination, which is essential for Akt membrane recruitment and its phosphorylation at T308 and S473 [32]. This study further extends the roles of TRAF6 in cell survival and oncogenic signaling.

In addition to a well-established role of TRAF6 in IL-1R/TLR-mediated signaling pathways during inflammation, a recent report has also implicated TRAF6 in muscle atrophy induced by denervation or cachexia [33]. However, not much is known about its role in myogenesis. In the current study, we showed that TRAF6 is specifically involved in myogenic differentiation. Knockdown of TRAF6 in myoblasts inhibited myogenic differentiation in a p38 MAPK- and Akt-dependent manner. Deliberate activation of either pathway rescued the differentiation defect caused by TRAF6 knockdown, suggesting that both pathways mediate the myogenic effect of TRAF6. Consistently, TAK1 was also found to function downstream of TRAF6 in myogenic differentiation. When TRAF6 was knocked down in vivo, it also inhibited the injury-induced muscle regeneration. Collectively, our work showed that TRAF6 promotes myogenic differentiation and muscle regeneration.

## Results

### Knockdown of TRAF6 inhibits myogenic differentiation

**Figure 1:**
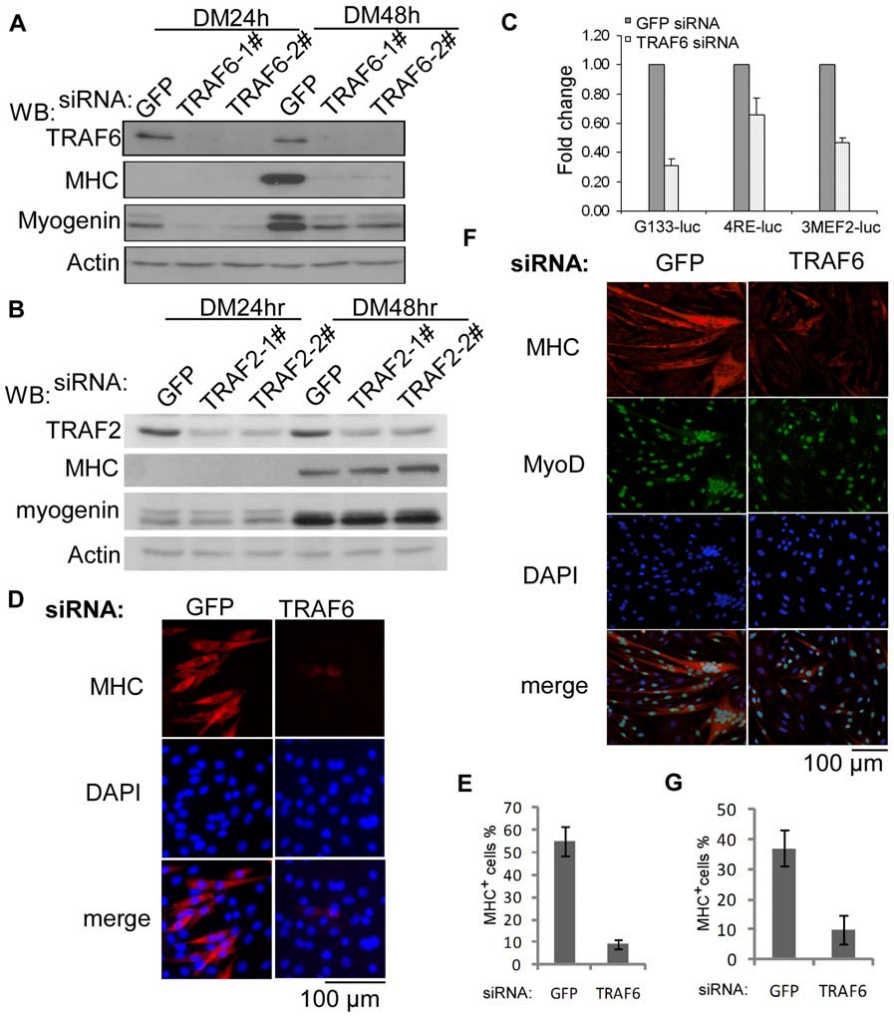
TRAF6 was required for myogenic differentiation. (A, B) C2C12 cells were transfected with a GFP-siRNA, two different TRAF6-siRNAs, or two different TRAF2-siRNAs as indicated. (C) C2C12 cells were transfected in triplicate with either the GFP-siRNA or TRAF6-siRNA together with different luciferase reporter constructs as indicated. Twenty-four hours (h) after transfection, cells were induced to differentiate for 24 h or 48 h before harvest. For (A, B), 60 µg of whole cell extracts (WCE) were subjected to SDS-PAGE and Western blot (WB) analysis for various proteins indicated on the left side of the panels. For (C), cells were harvested after 24 h in DM and WCE were prepared and subjected to luciferase assays. Fold change was calculated as the ratio of the luciferase activity of the TRAF6-siRNA transfected cells over that of the GFP-siRNA transfected cells. The results were presented as mean+s.d. (D–G) C2C12 cells (D, E) or primary myoblasts (F, G) were transfected with the GFP-siRNA or TRAF6-siRNA. Cells were fixed at DM 48 h and subjected to immunostaining for myosin heavy chain (MHC) or MyoD. The nuclei were counterstained with DAPI. In (E, G), the percentage of MHC-positive cells was calculated as the ratio of the number of nuclei in MHC-positive cells over that of DAPI-positive nuclei (E) or MyoD-positive nuclei (G). Cells from five different microscopic fields were counted and the results were presented as mean+s.d.

**Figure 2:**
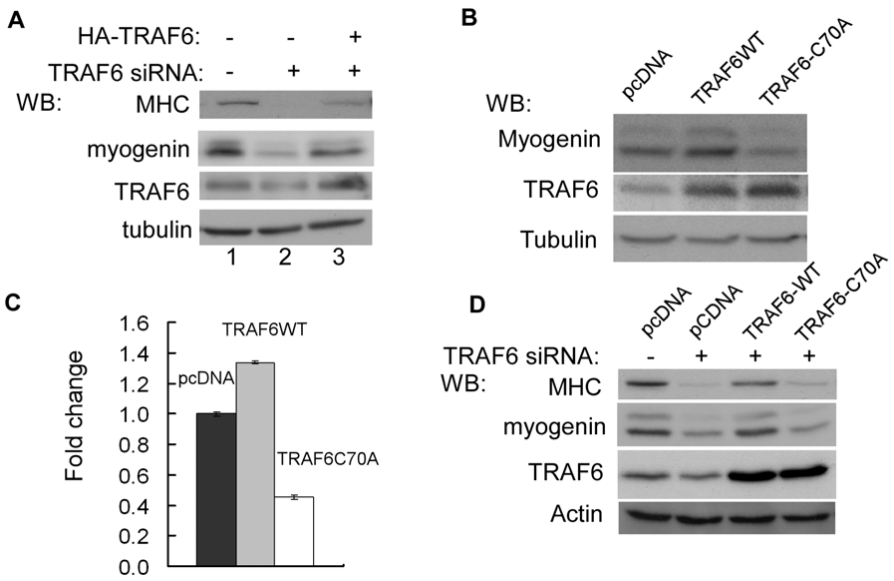
The E3 ligase activity of TRAF6 was required for myogenic differentiation. (A) C2C12 cells were transfected with either the GFP-siRNA or the TRAF6-siRNA together with an empty vector or a vector encoding human TRAF6 as indicated. (B) C2C12 cells were transfected with either an empty vector (pcDNA) or an expression vector encoding either the wild-type (WT) or an E3 ligase-defective mutant TRAF6. (C) C2C12 cells were transfected in triplicate with G133-*luc* together with pcDNA or an expression vector encoding the wild-type or the mutant human TRAF6. Twenty-four hours after transfection, cells were switched to DM for another 24 hours. WCEs were prepared and subjected to luciferase assays. Fold change was calculated as the ratio of the luciferase activity in TRAF6-transfected cells over that in pcDNA-transfected cells. The results were presented as mean+s.d. (D) C2C12 cells were transfected with the GFP-siRNA or TRAF6-siRNA together with pcDNA or an expression vector encoding the wild type or mutant human TRAF6. For (A), (B), and (D), cells were harvested at DM 36 h and WCE were subjected to SDS-PAGE and Western blot analysis for various proteins as indicated.

To explore potential roles of TRAF proteins in myogenic differentiation, we first focused on TRAF2 and TRAF6, the two best-characterized members in the TRAF family. Two specific siRNAs targeting different regions of either TRAF2 or TRAF6 genes were designed and used to transfect C2C12 cells. Cells were induced to differentiate and cellular proteins were prepared 24 or 48 hours after induction. As shown in Fig. 1A and 1B, both the TRAF2 and TRAF6 siRNAs were effective in knocking down their intended targets. However, only the TRAF6-siRNAs specifically inhibited the expression of myogenin and myosin heavy chain (MHC), two muscle-specific proteins frequently used as early and late differentiation markers respectively (Fig. 1A). In contrast, two TRAF2-siRNAs had no such effects (Fig. 1B). The TRAF6-siRNA also inhibited the activity of several myogenic luciferase reporter genes including G133-*luc*, a reporter driven by 133 base-pair mouse proximal *myogenin* promoter; 3×MEF2-*luc*, a MEF2-dependent reporter with three tandem repeats of a MEF2 site; and 4RE-*luc*, an MRF-dependent reporter with four tandem repeats of an E box (Fig. 1C) [11]. Our data suggested that TRAF6 is required for the normal myogenic function of MRFs and MEF2s as well as the expression of the *myogenin* gene. Consistently, by phase-contrast and fluorescent microscopy, we showed that knockdown of TRAF6 compromised the formation of multinucleated, MHC-positive myotubes in C2C12 cells (Fig. 1D, E). As C2C12 cells are immortalized myoblasts, to ascertain the myogenic role of TRAF6, we also knocked down TRAF6 in proliferating myoblasts freshly isolated from mouse muscles. These primary myoblasts were known to be MyoD-positive [34]. As shown in Fig. 1F and G, specific knockdown of TRAF6 in primary myoblasts inhibited their differentiation as evidenced by greatly reduced number of multinucleated, MHC-positive myotubes. To further exclude the possibility that TRAF6-siRNAs inhibited myogenic differentiation due to their potential “off-target” effects, we performed a “rescue” experiment. We first co-transfected C2C12 cells with a TRAF6-siRNA together with a cDNA expression vector encoding human TRAF6, which was resistant to the mouse TRAF6-siRNA due to the sequence difference in the siRNA targeting region (our unpublished data). As shown in Fig. 2A, human TRAF6 effectively rescued the inhibitory effect of the mouse TRAF6-siRNA as evidenced by re-elevated expression of myogenin and MHC (lane 3).

### The E3 ligase activity of TRAF6 is required for myogenic differentiation

Although TRAF6 is an established ubiquitin E3 ligase [35], it was unclear whether its E3 ligase activity was involved in myogenic differentiation or not. To address this issue, we first transfected C2C12 cells with an expression vector encoding either the wild-type or an E3 ligase-defective mutant TRAF6 (i.e., TRAF6-C70A) [32]. As shown in Fig. 2B, the two constructs had opposite effects: while the wild-type TRAF6 slightly enhanced the expression levels of myogenin, TRAF6-C70A reduced levels of myogenin compared to the control. This suggested that the mutant TRAF6 competed with the endogenous TRAF6 and compromised its function. Consistently, when co-transfected with G133-*luc*, the wild-type TRAF6 enhanced, while TRAF6-C70A inhibited, the activity of the reporter (Fig. 2C). Furthermore, we asked whether TRAF6-C70A could rescue the differentiation defect caused by the TRAF6-siRNA. We co-transfected C2C12 cells with either the GFP-siRNA or TRAF6-siRNA together with an empty vector or a TRAF6-C70A-expressing vector. As shown in Fig. 2D, TRAF6-C70A failed to rescue the differentiation defect caused by TRAF6 knockdown. Collectively, our data above suggested that the E3 ligase activity of TRAF6 is required during myogenic differentiation.

### TAK1 acts downstream of TRAF6 in myogenic differentiation

**Figure 3:**
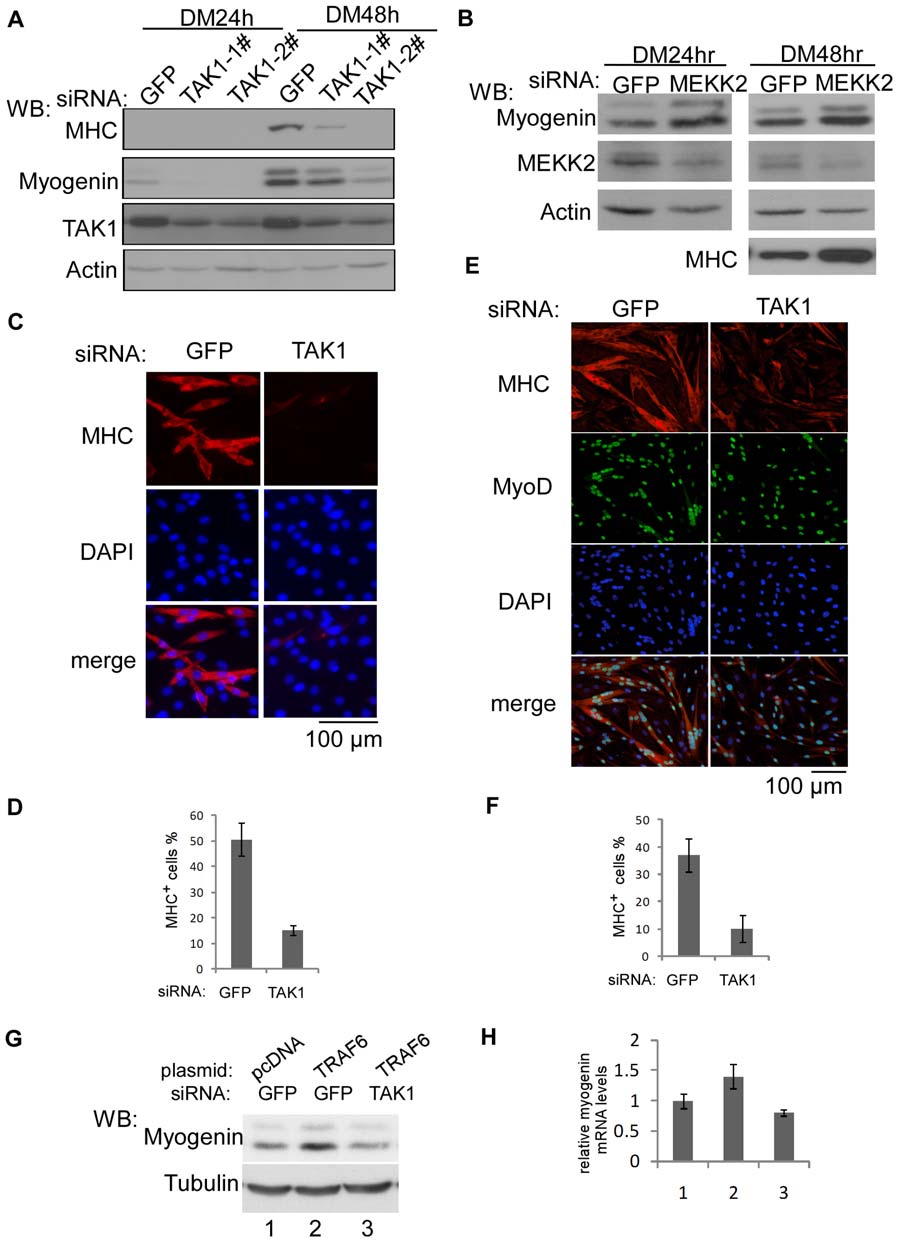
TAK1 was required for myogenic differentiation. (A, B) C2C12 cells were transfected with various siRNAs as indicated. Cells were harvested at the indicated time points and WCEs were subjected to SDS-PAGE and Western blot analysis. (C–F) C2C12 cells (C, D) or primary myoblasts (E, F) were transfected with either the GFP-siRNA or the TAK1-siRNA. Forty-eight hours after induction of differentiation, cells were fixed and subjected to immunostaining for MHC or MyoD. The nuclei were counterstained with DAPI. In (D, F), the percentage of MHC-positive cells was calculated the same way as that described in the legend of Fig. 1(E, G). (G, H) C2C12 cells were co-transfected with an empty vector or a TRAF6-expressing vector together with the GFP-siRNA or TAK1-siRNA as indicated. Twenty-four hours after induction of differentiation, cells were harvested for subsequent analysis: WCE were subjected to SDS-PAGE and Western blot analysis (G), while total RNA was prepared for RT-qPCR analysis (H) of relative myogenin gene expression. 1, 2, 3 in (G) and (H) denote the same set of samples.

To identify downstream mediators of TRAF6 in myogenic differentiation, we turned to TAK1, a member of the MAP2K kinase (MAP3K) family and a well-established downstream target of TRAF6 [25], [26]. As expected, we found that two siRNAs targeting different regions of the TAK1 gene could effectively inhibit myogenic differentiation as evidenced by decreased expression of myogenin and MHC (Fig. 3A). As a control, we also designed an siRNA targeting MEKK2, another member of the MAP3K family [36]. As shown in Fig. 3B, the MEKK2-siRNA did not inhibit the expression levels of myogenin or MHC. In fact, the MEKK2-siRNA even slightly promoted the expression of both myogenin and MHC (Fig. 3B). Consistently, when a TAK1-siRNA was transfected into C2C12 cells or primary myoblasts, it greatly decreased the number and size of MHC-positive myotubes (Fig. 3C–F). To test whether TAK1 functions downstream of TRAF6, we transfected C2C12 cells with a TRAF6-expressing plasmid with or without the TAK1-siRNA. Consistent with the data in Fig. 2B, TRAF6 enhanced the expression of myogenin at both the protein and mRNA levels (Fig. 3G, H). However, this stimulatory effect was abrogated when TAK1 was knocked down. Collectively, our data showed that TAK1 is a key downstream mediator of TRAF6 involved in myogenic differentiation.

### The kinase activity of TAK1 is required for myogenic differentiation

**Figure 4:**
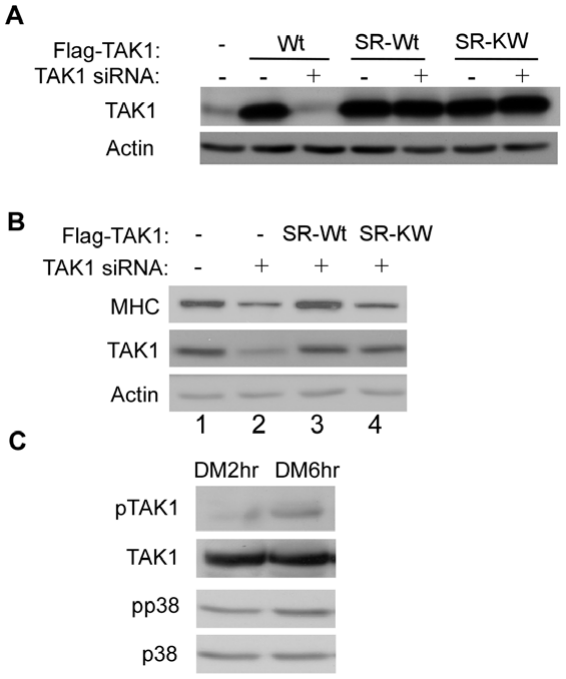
The kinase activity of TAK1 was required for myogenic differentiation. (A, B) C2C12 cells were co-transfected with a control siRNA or TAK1-siRNA together with various expression vectors as indicated. Wt: Flag-TAK1; SR-Wt: an siRNA-resistant wild-type TAK1; SR-KW: an siRNA-resistant kinase-dead TAK1. Cells were harvested at DM 36 h. The minus sign means that a control siRNA or an empty vector was used. (C) C2C12 cells were induced to differentiate in DM for 2 to 6 hours. WCE from (A–C) were prepared and subjected to SDS-PAGE and Western blot analysis for various proteins as indicated.

Although our data above showed that TAK1 is required for myogenic differentiation, it remained unclear whether its kinase activity is required in this process or not. To address this issue, we first generated two siRNA-resistant TAK1 expression constructs: one encoding the wild-type protein (i.e. SR-Wt), and the other encoding a kinase-dead mutant (i.e. SR-KW) [37]. We then transfected C2C12 cells with the TAK1-siRNA together with one of the two siRNA-resistant TAK1 constructs. As shown in Fig. 4A, both TAK1 constructs were expressed at similar levels and were indeed resistant to the TAK1-siRNA. However, only the wild-type TAK1 (lane 3), but not the kinase-dead TAK1 (lane 4), was able to effectively rescue the reduction in MHC levels caused by the TAK1-siRNA (Fig. 4B). Moreover, using an antibody specifically recognizing the autophosphorylated form of TAK1 (i.e., the active TAK1) [38], we showed that the levels of the active TAK1 in C2C12 cells indeed increased upon myogenic differentiation (Fig. 4C), which paralleled an increase in the levels of the active p38 MAPK in differentiating myoblasts. This result was consistent with the data from the “rescue” experiment.

### Both p38 MAPK and Akt mediate the effect of TRAF6 during myogenic differentiation

**Figure 5:**
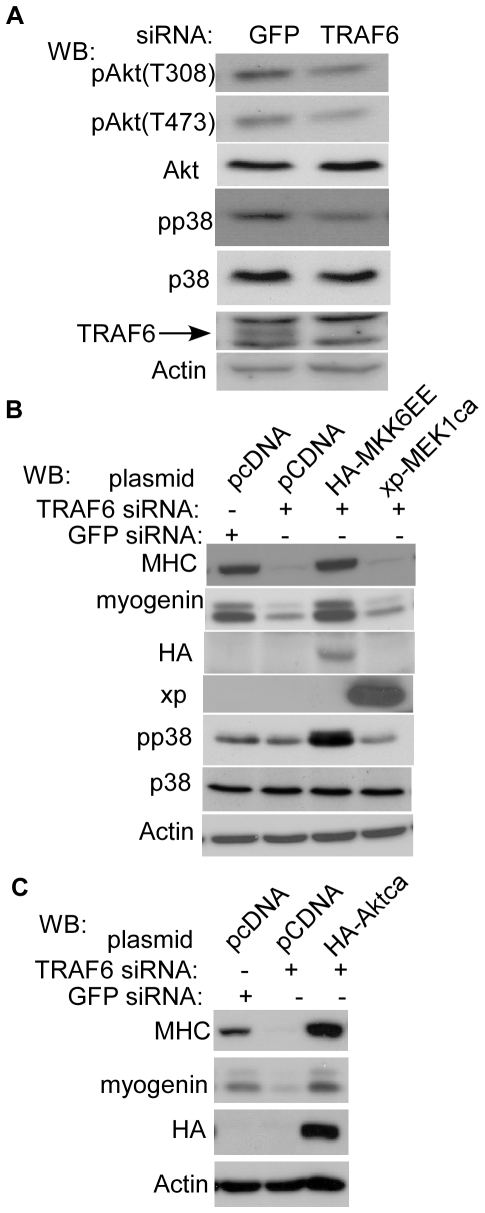
Both p38 MAPK and Akt mediated the effect of TRAF6 during myogenic differentiation. (A–C) C2C12 cells were transfected with the GFP-siRNA or TRAF6-siRNAs with or without various cDNA expression vectors as indicated. (A). Cells were harvested 24 hours after transfection. (B, C) Twenty-four hours after transfection, cells were induced to differentiate in DM for another 36 hours before harvest. WCEs from (A–C) were subjected to SDS-PAGE and Western blot analysis for various proteins as indicated. MKK6EE, MEK1ca and Aktca are the constitutively active mutants of MKK6, MEK1, and Akt respectively. xp: Xpress-tag.

To identify intracellular signaling pathways activated by TRAF6 in myogenic differentiation, we first turned to p38 MAPK and Akt which are known to be activated by TRAF6 [32], [39]. In the first set of experiments, we studied the impact of TRAF6 knockdown on the activation status of Akt and p38 MAPK. As shown in Fig. 5A, the siRNA-mediated TRAF6 knockdown reduced the levels of Akt phosphorylation at Thr308 and Ser473, which are required for maximal Akt activation [40]. Moreover, knockdown of TRAF6 also decreased the levels of the phosphorylated (i.e., active) p38 MAPK. In the second set of experiments, we tested whether deliberate activation of either p38 MAPK or Akt could rescue the differentiation defect caused by the TRAF6-siRNA. To do so, we co-transfected C2C12 cells with the TRAF6-siRNA together with an expression vector encoding MKK6EE that constitutively activated p38 MAPK, MEK1ca that constitutively activated ERK, or a constitutively active Akt (i.e., myristoylated Akt). As shown in Fig. 5B and 5C, only the constitutively active MKK6EE (Fig. 5B) and Akt (Fig. 5C) effectively reversed the inhibitory effect of the TRAF6-siRNA based on re-elevated expression of myogein and MHC, while the constitutively active MEK1ca had no such effects (Fig. 5B, lane 4). Taken together, our data above suggested that both p38 MAPK and Akt mediated the effect of TRAF6 during myogenic differentiation.

### TRAF6 knockdown impairs the injury-induced muscle regeneration

**Figure 6:**
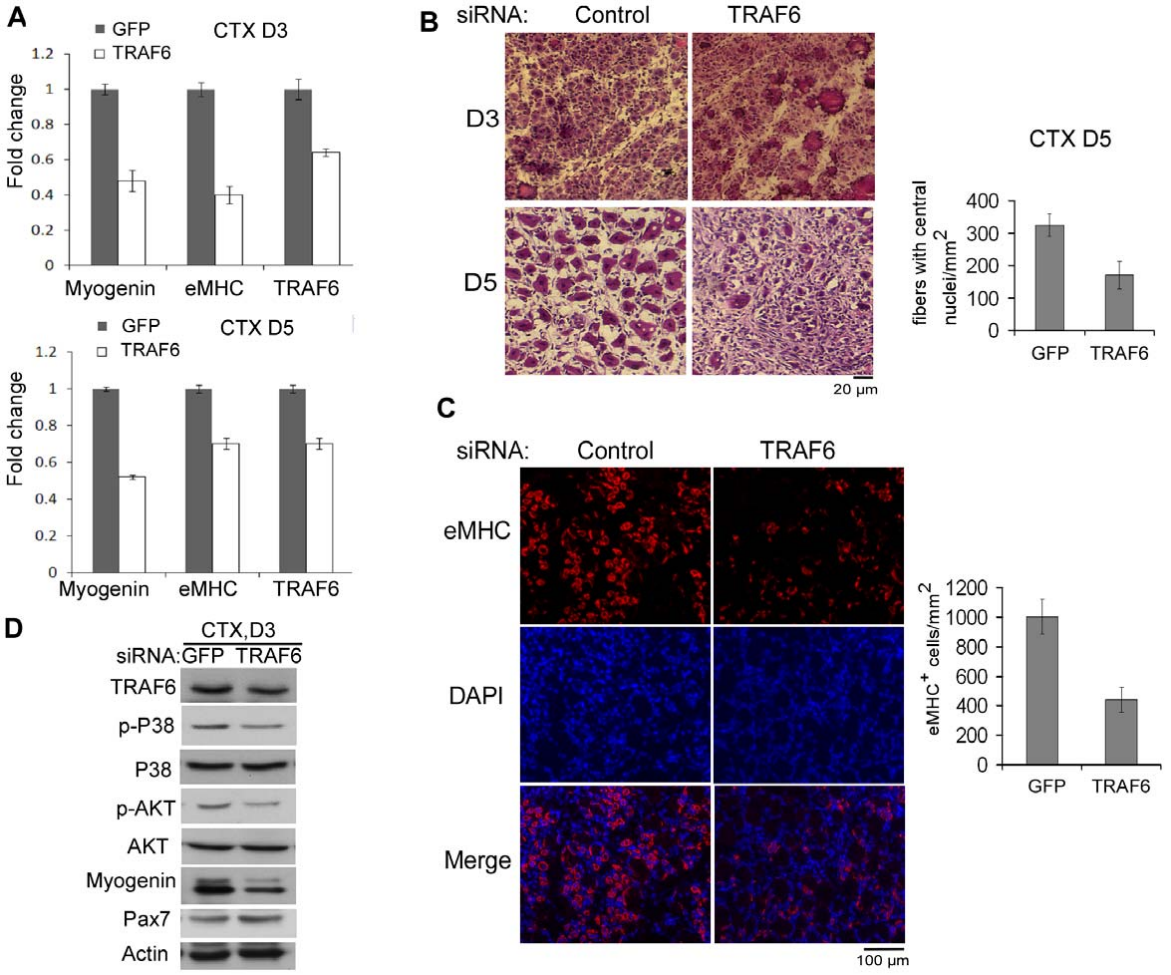
Knockdown of TRAF6 impaired the CTX-induced muscle regeneration. (A–D) Tibialis anterior (TA) muscles of adult mice were injected with 30 µl of 10 µM CTX. One day after CTX injection, the GFP-siRNA and TRAF6-siRNA mixed with liposome were injected into the left and right injured TA muscles respectively once per day. TA muscles were collected at day (D) 3 and day 5 after CTX injection. The total RNA, muscle sections, or WCEs were prepared and subjected to RT-qPCR (A), hematoxylin-eosin staining (B), immunostaining (C), or SDS-PAGE and Western blot analysis (D). The number of myofibers with central nuclei (B) or the number of eMHC-positive myofibers (C) was calculated from five different microscopic fields. The results were presented as mean+s.d. CTX: cardiotoxin. eMHC: embryonic MHC.

To further explore the myogenic role of TRAF6 in vivo, we resorted to the cardiotoxin (CTX)-induced muscle injury and regeneration model [34]. The GFP-siRNA or TRAF6-siRNA mixed with liposome was directly injected into the CTX-injured tibialis anterior (TA) muscles of adult mice followed by electroporation for multiple times. Based on a fluorescent dye-labeled control siRNA, we showed that this method could efficiently deliver siRNA into TA muscles (Fig.S1A). The TA muscles were dissected out at different time points to monitor the progress of the regeneration. As shown in Fig. 6A, at both day 3 and day 5 after injury, the TRAF6-siRNA could effectively reduce the TRAF6 mRNA levels by about 40%. The mRNA levels of both *myogenin* and embryonic *MHC* (*eMHC*) were also greatly reduced by the TRAF6-siRNA (Fig. 6A). Consistently, we found that the number of newly-formed regenerating myofibers, which were characterized by either centrally-localized nuclei or expression of eMHC [34], was also reduced in TA muscles injected with the TRAF6-siRNA (Fig. 6B, 6C). By Western blotting, we confirmed that TRAF6 knockdown in muscles reduced the expression levels of myogenin as well as the levels of the active p38 MAPK and Akt (Fig. 6D). In contrast, the expression levels of Pax7 slightly increased. To find out whether the defective muscle regeneration was due to an inhibitory effect of the TRAF6-siRNA on myoblast proliferation, we examined the Pax7-positive muscle precursor cells (MPC) (including muscle satellite cells and proliferating myoblasts) in regenerating muscles 3 days after injury. Interestingly, we found that TRAF6 knockdown increased the number of Pax7-positive MPC (Fig.S1B). To further ascertain this effect, we knocked down TRAF6 in isolated primary myoblasts. We found that TRAF6 knockdown indeed promoted myoblast proliferation as evidenced by an increase in the number of Pax7-positive cells as well as BrdU-positive cells (Fig.S1C, 1D). As TRAF6 is expressed in macrophages, it was possible that TRAF6 knockdown could inadvertently affect macrophage infiltration, which is a critical early step in muscle regeneration [41]. As macrophage infiltration peaked in the first two days after injury, we examined CD68-positive macrophages in regenerating muscles 2 days after injury. We found that TRAF6 knockdown did not affect macrophage infiltration (Fig.S2A, 2B). Consistently, by quantitative RT-PCR analysis, we showed that the mRNA levels of CD68 and F4/80, both of which are unique markers of macrophages, were not affected by TRAF6 knockdown (Fig.S2C). Our data above indicated that TRAF6 is required for muscle regeneration mainly by promoting myoblast differentiation.

## Discussion

The involvement of the p38 MAPK pathway in myogenic differentiation has been well documented. Multiple myogenic substrates of p38 MAPK have been identified that could provide some molecular basis for an indispensable role of p38 MAPK in myogenesis [14], [15]. In contrast to what we already know about various downstream targets of p38 MAPK, very little is known about its upstream activators during myogenic differentiation. Our findings here show that TRAF6 is a key p38 activator in myogenic differentiation. Interestingly, TRAF2, another related member of the TRAF family proteins, has no such pro-myogenic effect in our assays, suggesting that the pro-myogenic effect of TRAF6 is quite specific. Our results are consistent with a recent report that also implicates TRAF6 in myogenic differentiation [42]. However, no underlying mechanism was provided in that report. We showed here that TRAF6 promotes myogenic differentiation mainly by activating the TAK1/p38 MAPK pathway and the Akt pathway, as knockdown of TRAF6 reduced the levels of active p38 MAPK and Akt in myoblasts (Fig. 5). Importantly, deliberate activation of either the p38 MAPK pathway or Akt pathway could rescue the differentiation defect caused by TRAF6 knockdown. As an ubiquitin E3 ligase, TRAF6 is known to be required for activation of both TAK1 and Akt via distinct mechanisms. For TAK1, TRAF6 generates unanchored K63-linked polyubiquitin chain that is bound by TAB2 leading to TAK1 activation [30]. For Akt, TRAF6 catalyzes direct K63-linked polyubiquitination of Akt to result in its activation [32]. Interestingly, TAK1 was also shown to promote Akt activation during myoblast differentiation [24]. This effect is most likely indirect, as lack of TAK1 did not affect the IGF1-induced Akt activation in myoblasts. Therefore, the molecular basis for the involvement of TAK1 in Akt activation remains to be elucidated.

Other than TRAF6, another well-established upstream p38 activator is Cdo, a cell surface protein known to trigger activation of p38 MAPK in myoblasts in response to N-cadherin ligation during myogenic differentiation [43]. Bnip-2, a scaffold protein for Cdc42, and JLP, a scaffold molecule for p38 MAPK, were found to be key signal transducers in mediating the pro-myogenic effect of Cdo [44], [45]. It is unclear whether TRAF6 is involved in the N-cadherin/Cdo-mediated p38 MAPK pathway during myogenic differentiation. It is likely that the TRAF6/TAK1 pathway and the N-cadherin/Cdo pathway are two parallel but non-redundant pathways, both leading to p38 activation in response to different signals. More future work is needed to study potential cross-talk between these two pathways.

The involvement of the pathway consisting of TRAF6/TAK1/MKK3/6/p38 MAPK in myogenic differentiation further suggests that the potential extracellular stimuli that trigger myoblast differentiation in vivo may belong to the family of pro-inflammatory ligands that utilize IL-1R/TLR. This hypothesis is particularly relevant during injury-induced muscle regeneration in adult animals, as such pro-inflammatory molecules are quickly induced and abundantly expressed in damaged muscles. Consistently, in the CTX-induced muscle regeneration model, we showed that knockdown of TRAF6 by siRNA greatly compromised the regeneration process (Fig. 6B, C). Interestingly, the proliferation of the Pax7-positive muscle precursor cells (MPC) was not inhibited by TRAF6 knockdown. In contrast, TRAF6 knockdown slightly promoted expansion of the Pax7-positive MPC (Fig.S1). This is most likely due to the reduced p38 activity, which has been causally linked with enhanced proliferation of the Pax7-positive MPC [46]. This result further strengthens our view that the regeneration defect caused by TRAF6 knockdown is mainly due to its role in myoblast terminal differentiation instead of proliferation of MPC. Moreover, although we are mainly concerned with the role of TRAF6 in myoblasts here, we cannot completely rule out a positive role of TRAF6 in inflammatory cells, especially macrophages that are known to be indispensable for muscle regeneration [41]. A quick survey of injured muscles showed that TRAF6 knockdown did not affect macrophage infiltration into the injured muscles (Fig.S2). Nevertheless, TRAF6 in macrophages could still indirectly contribute to muscle regeneration by regulating the expression of certain secretable factors (e.g. cytokines and chemokines) that in turn may regulate muscle satellite cells. In the future, the use of conditional knockout mice with TRAF6 specifically deleted in myeloid cells may reveal additional role of TRAF6 in these inflammatory cells during muscle regeneration.

Our findings here could have potential therapeutic application. One can envision that small molecules that trigger the activation of the TRAF6/TAK1/p38 MAPK pathway and TRAF6-Akt pathway are likely beneficial in promoting muscle regeneration in response to various injuries.

## Materials and Methods

### Animal handling

All C57BL/6J mice were maintained and handled in accordance with the protocols approved by the Animal Ethics Committee of the Hong Kong University of Science & Technology and the Department of Health of the Hong Kong Special Administrative Region [Ref No: (11-28) in DH/HA&P/8/2/2 Pt.3].

### Cell line, DNA constructs and reagents

C2C12 mouse myoblasts (American Type Culture Collection, Manassas, VA) were cultured in Dulbecco's modified Eagle's medium (DMEM) supplemented with 20% fetal bovine serum (FBS), 100 units/ml penicillin, and 100 µg/ml streptomycin (growth medium, or GM) in a humidified incubator at 37°C with 5% CO_2_. To induce differentiation, near-confluent C2C12 cells were grown in DMEM supplemented with 2% horse serum (differentiation medium, or DM). G133-luc, 4RE-luc, 3MEF2-luc HA-MKK6EE, HA-TRAF6, Flag-TAK1(wt), and the kinase-dead Flag-TAK1(KW) were described previously [17], [47], [48]. Silent mutation was introduced into Flag-TAK1(wt) and Flag-TAK1(KW) constructs to generate the siRNA-resistant Flag-TAK1(SR-Wt) and Flag-TAK1(SR-KW) respectively using the following oligonucleotide: 5′- CGA GGA AAT TGA TTA CAA GGA GAT CGA GGT GGA AGA GG -3′ (top strand). TRAF6-C70A was a gift from Dr. Huikuan Lin (M.D. Anderson Cancer Center, Houston, TX). 4′,6-diamidino-2-phenylindole (DAPI) and cardiotoxin (CTX) were from Sigma (Sigma-Aldrich, St.Louis, MO) and Latoxan (Latoxan, Valence, France) respectively. All DNA recombinant work follows the National Institutes of Health guidelines.

### siRNA transfection

The following siRNAs were used: EGFP-siRNA (5′ GCTGACCCTGAAGTTCATC 3′); TAK1-siRNAs (1# 5′ GAGATCGACTACAAGGAGA 3′; 2# 5′ CCATTATAACAGTTCATGA 3′); MEKK2-siRNAs (1# 5′ GCACTAGTAGTGGAGGCAG 3′; 2# 5′ CTGAGAACGTGACGAGGAA 3′); and TRAF6 (1# 5′ ACCACGAAGAGGTCATGGA 3′; 2# 5′ TAAGCCAACCAGTTACTTT 3′). siRNAs were synthesized at either Dharmacon Inc. or RiboBio Co, Ltd (Guangzhou, China). A fluorescent dye-labeled control siRNA (Cy3-Ncontrol) used to monitor the transfection efficiency was purchased from RiboBio Co, Ltd. To deliver oligonucleotide-based siRNAs into cells, 40–60% confluent C2C12 cells were transfected with 20 nM siRNA using lipofectamine RNAiMAX (Invitrogen, Carlsbad, CA). Briefly, for 3.5 cm dishes, siRNAs were diluted in 100 µl of Optimal DMEM and then 4 µl of lipofectamine RNAiMAX was added directly into the diluted siRNA. The mixture was left to stand at room temperature for 20 min before being added to cells.

### Transient transfection of plasmids and cell lysis

Transient transfection was performed using Lipofectamine/PLUS Reagents (Invitrogen) according to the manufacturer's instruction. Briefly, 6 µl of PLUS Reagent was mixed with 1 µg of DNA diluted in 100 µl of DMEM without serum and antibiotics for 15 min at room temperature. Then 100 µl of DMEM containing 4 µl of Lipofectamine was added to it and incubated for another 15 min. The mixture was added to cells in 3.5 cm culture dishes, and incubated at 37°C for 3 hours before being removed and replaced with normal growth medium. Cells were lysed on ice for 10 min in the lysis buffer (50 mM Hepes at pH 7.6, 10% glycerol, 1% Triton X-100, 150 mM NaCl, 1 mM EGTA, 1.5 mM MgCl_2_, 100 mM NaF, 20 mM PNPP, 20 mM β-glycerol phosphate, 2 mM dithiothreitol (DTT), 50 µM sodium vanadate, 0.5 mM phenylmethylsulfonyl fluoride, 2 µg/ml aprotinin, 0.5 µg/ml leupeptin, 0.7 µg/ml pepstatin), followed by removal of insoluble debris in a bench-top centrifuge at 12,000× g for 2 min to obtain soluble whole cell extracts (WCE).

### Antibodies, immunostaining, and Western blotting

The following antibodies were from Santa Cruz Biotechnology (Santa Cruz, CA): anti-myogenin (F5D), anti-β-actin, anti-MyoD (M-318), anti-MEF2 (c-21), anti-TAK1(M-579), and anti-TRAF6 (D-10). Anti-total p38, anti-phospho-p38 (Thr180/Tyr182), anti-phospho-TAK1 (Thr187), anti-phospho-JNK (Thr183/Tyr185), anti-total JNK, anti-phospho-ERK(Thr202/Tyr204), and anti-total ERK were purchased from Cell Signaling Inc (Danvers, MA). Anti-CD68 was purchased from AbD Serotec (Oxford, UK). For immunostaining, cells were first fixed in 4% paraformaldehyde for 15 min, then permeabilized in 0.2% Triton X-100 for 15 min and blocked in 5% BSA in PBS for 1 hour. Cells were then incubated with a primary antibody overnight, washed three times with PBS, and re-incubated with a Rhodamine- or Fluorescein-conjugated secondary antibody (Jackson ImmunoResearch Laboratories Inc., West Grove, PA) for one hour. 100 ng/ml of DAPI was then added for another 10 minutes to stain the nuclei. Fluorescence microscopy was performed using an Olympus IX70 microscope linked to a charge-coupled device digital camera (Spot RT, Diagnostic Instruments Inc., Sterling Heights, MI). Western blotting was performed as described previously [11].

### Luciferase reporter assays

Cell lysates (10 µl) were mixed with 150 µl of the assay buffer (0.1 M Tris-acetate, pH 7.8, 1 mM EDTA, 10 mM Mg(OAc)_2_) supplemented with 66 µM luciferin and 2 mM ATP. Total light emission was measured in an LB9507 luminometer (EG&G Berthold). The luciferase units of each sample were normalized against the protein concentration determined by a protein assay reagent (BioRad).

### Preparation of mouse primary myoblasts

We followed a protocol from Dr. G Pavlath's laboratory. Briefly, Two month-old C57BL/6J mice were sacrificed by cervical dislocation and then rinsed with 70% ethanol. All the limb muscles were dissected away from the skins and bones, minced into fine pieces with scissors, and transferred to 40 ml PBS in a 50 ml centrifuge tube. After mixing followed by centrifugation at 300× g for 4 min, the supernatants with blood and fat were removed. 10 ml of DMEM and 1 ml of 1% pronase were added to the tube containing minced tissues. The tube was incubated at 37°C with continuous shaking for 1.5 hours. After centrifugation at 300× g for 5 min, the enzyme solution was removed, and the pellets were resuspended in 5∼10 ml DMEM, and triturated with a pipet to loosen cells. The supernatants were passed through a 100 µm filter. The myoblasts were pelleted by centrifugation at 300× g for 5 min and resuspended in 10 ml of growth media (F10+20% FBS+5 ng/ml bFGF) in non-coated plates. Fibroblasts were allowed to “settle down” for 45∼60 mins. The floating myoblasts in media were transferred to a Matrigel (BD Biosciences)-coated plate. The growth media were changed every 36 hours. Myoblasts were induced to differentiate in DM (DMEM with 5% horse serum).

### Delivery of siRNA into skeletal muscles and hematoxylin-eosin staining

Tibialis anterior (TA) muscles of 6–8 week-old C57BL/6J mice were injected with 30 µl of 10 µM CTX. One day after the injury, 6 µl of 50 µM GFP-siRNA or TRAF6-siRNA in OPTI-MEDIUEM was mixed with 6 µl of RNAiMAX (Invitrogen) and incubated for 15 min at room temperature. The GFP-siRNA and TRAF6-siRNA were directly injected into the left and right TA muscles of the same mice respectively followed by electroporation using a BTX ECM 830 generator (mode: LV; field strength: 175 V/cm; pulse length: 20 ms; number of pulses: 8) and a pair of 7-mm Tweezertrodes (BTX) with one electrode attached to the TA muscle and the other to the gastrocnemius muscle of the lower hind limb. The siRNA injection/electroporation was repeated once per day for various days before mice were sacrificed and TA muscles were collected. The isolated TA muscles were subjected to either total RNA purification or fixed in 4% paraformaldehyde and sectioned at 6∼8 µm on a cryostat (Leica) and then stained with hematoxylin and eosin.

### Quantitative RT-PCR (RT-qPCR)

cDNA was generated from total RNA by ImPromega-II reverse Transcription System from Promega. Power SYBR Green PCR Master Mix (Applied Biosystems) was used for real-time PCR. Primers used were as follows: TRAF6 (forward: 5′- AAAGCGAGAGATTCTTTCCCTG-3′; reverse: 5′-ACTGGGGACAATTCACTAGAGC-3′); myogenin (forward: 5′-GCAATGCACTGGAGTTCG-3′; reverse: 5′-ACGATGGACGTAAGGGAGTG-3′); CD68 (forward: 5′-ACAGGCAGCACAGT GGACATTC-3′; reverse: 5′-ATGAGAGGCAGCAAGAG-3′); F4/80 (forward: 5′-CCCAGCTTATGCCACCTGCA-3′; reverse: 5′-TCCAGGCCCTGGAACATTGG-3′); and the embryonic MHC (eMHC) (forward: 5′-AAAAGGCCATCACTGACGC-3′; reverse: 5′-CAGCTCTCTGATCCGTGTCTC-3′). The qPCR reaction was performed in a 7500 Fast Real-time PCR System (Applied Biosystems). More than 40 cycles of amplification were performed, with each consisting of a denaturation (95°C; 3 s) and an annealing/extension (60°C; 30 s) step. Data acquisition and the analysis of the qPCR assays were performed using the 7500 software (Version 2.0.2; Applied Biosystems).

## Supporting Information

Figure S1 **TRAF6 knockdown promoted proliferation of Pax7-positive muscle precursor cells.** (A) A Cy3-labeled control siRNA was mixed with liposome and injected into TA muscles. Muscle sections were prepared one day or two days after injection and subjected to fluorescent microscopy. The images were taken with a 4× objective. (B) TA muscles of adult mice were injected with CTX followed by treatment with the GFP-siRNA and TRAF6-siRNA as described in the legend of Fig. 6. TA muscles were collected at day 3 after CTX injection. Muscle sections were prepared and subjected to immunostaining for Pax7. The nuclei were counterstained with DAPI. The number of Pax7-positive cells was calculated from five different microscopic fields and the results were presented as mean+s.d. (C, D) Primary myoblasts were transfected with either the GFP-siRNA or TRAF6-siRNA. Twenty-four hours after transfection, cells were either left untreated (C) or treated with BrdU (D) for 1.5 hour before fixation. Cells were then subjected to immunostaining for Pax7, MyoD, and BrdU. The percentage of the Pax7-positive cells and the BrdU-positive cells was calculated as the ratio of the number of Pax7-positive nuclei (C) and BrdU-positive nuclei (D) over that of DAPI-positive nuclei. Cells from five different microscopic fields were counted and the results were presented as mean+s.d.
(TIF)

Figure S2 **TRAF6 knockdown did not affect macrophage infiltration.** (A–C) TA muscles of adult mice were injected with CTX followed by treatment with the GFP-siRNA and TRAF6-siRNA as described above in the legend of Fig.S1. TA muscles were collected at day 2 after CTX injection. Non-injured TA muscles were used as a control. Muscle sections or total RNA were prepared and subjected to immunostaining for CD68 (A, B) or RT-qPCR analysis for relative mRNA expression of CD68, F4/80, and TRAF6 (C). Images in (A) were taken with a 4× objective, while the same images in (B) were taken with a 20× objective. The nuclei were counterstained with DAPI.
(TIF)
