# Supplementary figures and images for: TRAF6 Promotes Myogenic Differentiation via the TAK1/p38 Mitogen-Activated Protein Kinase and Akt Pathways
Source: PLoS One. 2012 Apr 4;7(4):e34081. doi: 10.1371/journal.pone.0034081 (PMC3319550; doi:10.1371/journal.pone.0034081)

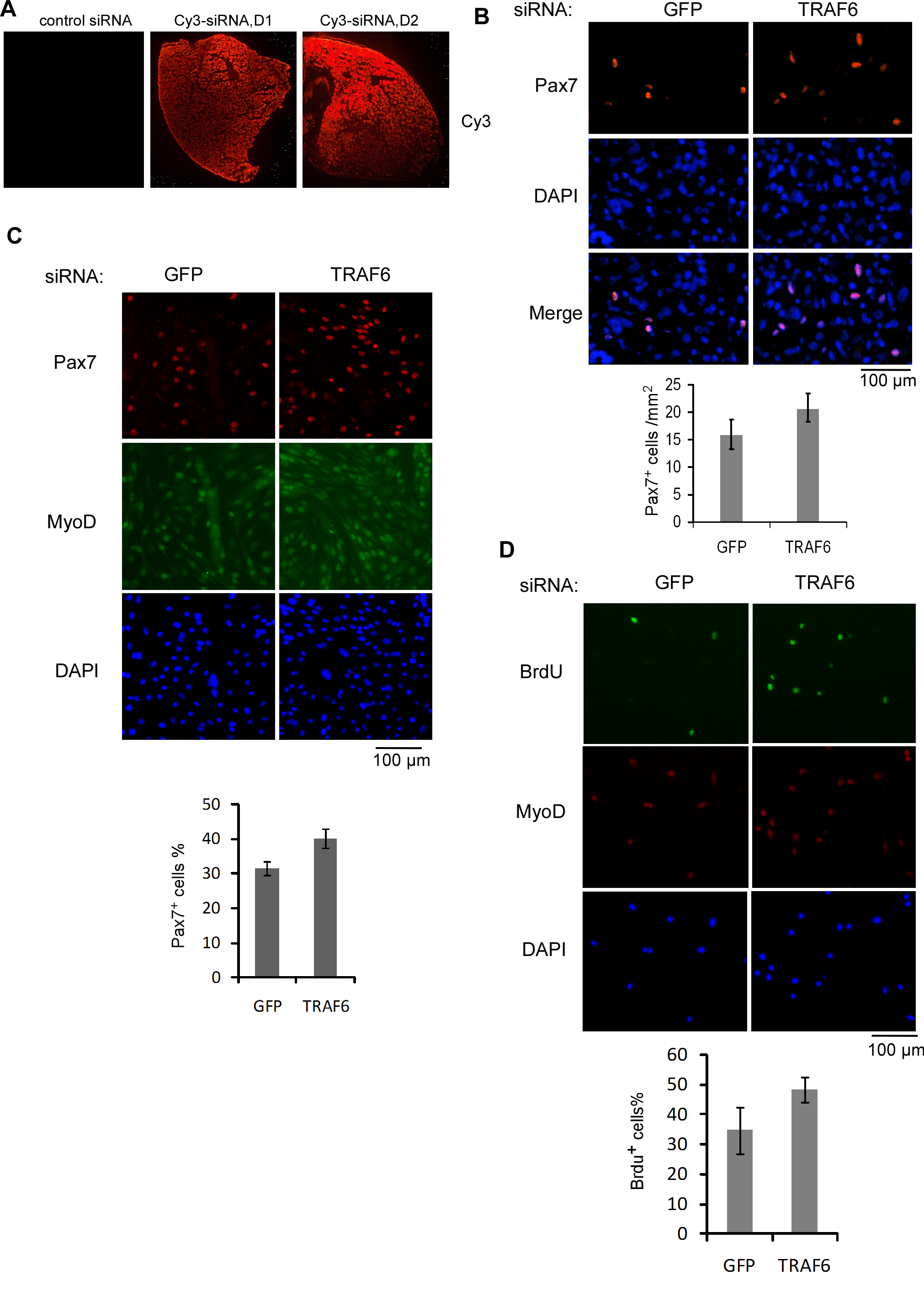

Supplement: Figure S1 — TRAF6 knockdown promoted proliferation of Pax7-positive muscle precursor cells. (A) A Cy3-labeled control siRNA was mixed with liposome and injected into TA muscles. Muscle sections were prepared one day or two days after injection and subjected to fluorescent microscopy. The images were taken with a 4× objective. (B) TA muscles of adult mice were injected with CTX followed by treatment with the GFP-siRNA and TRAF6-siRNA as described in the legend of Fig. 6. TA muscles were collected at day 3 after CTX injection. Muscle sections were prepared and subjected to immunostaining for Pax7. The nuclei were counterstained with DAPI. The number of Pax7-positive cells was calculated from five different microscopic fields and the results were presented as mean+s.d. (C, D) Primary myoblasts were transfected with either the GFP-siRNA or TRAF6-siRNA. Twenty-four hours after transfection, cells were either left untreated (C) or treated with BrdU (D) for 1.5 hour before fixation. Cells were then subjected to immunostaining for Pax7, MyoD, and BrdU. The percentage of the Pax7-positive cells and the BrdU-positive cells was calculated as the ratio of the number of Pax7-positive nuclei (C) and BrdU-positive nuclei (D) over that of DAPI-positive nuclei. Cells from five different microscopic fields were counted and the results were presented as mean+s.d. (TIF) [file pone.0034081.s001.tif]

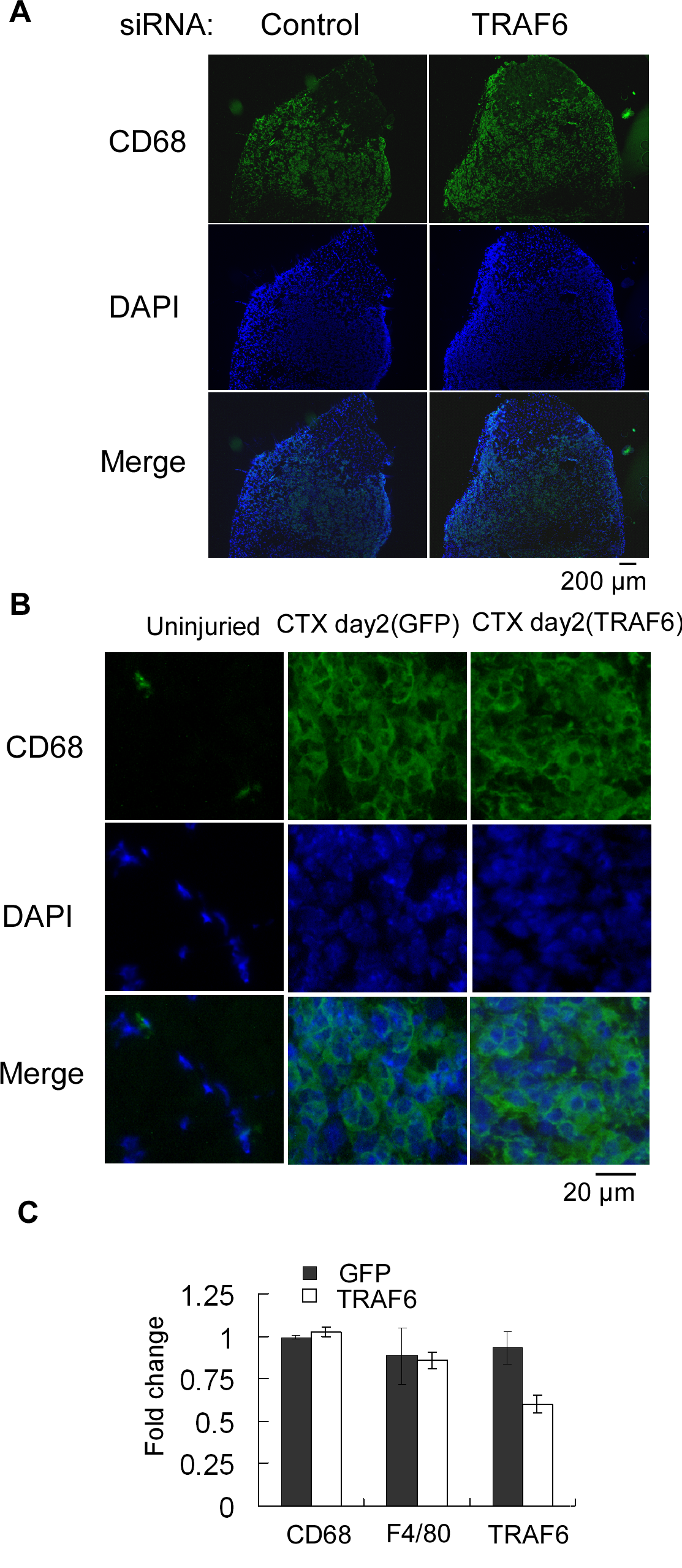

Supplement: Figure S2 — TRAF6 knockdown did not affect macrophage infiltration. (A–C) TA muscles of adult mice were injected with CTX followed by treatment with the GFP-siRNA and TRAF6-siRNA as described above in the legend of Fig.S1. TA muscles were collected at day 2 after CTX injection. Non-injured TA muscles were used as a control. Muscle sections or total RNA were prepared and subjected to immunostaining for CD68 (A, B) or RT-qPCR analysis for relative mRNA expression of CD68, F4/80, and TRAF6 (C). Images in (A) were taken with a 4× objective, while the same images in (B) were taken with a 20× objective. The nuclei were counterstained with DAPI. (TIF) [file pone.0034081.s002.tif]
